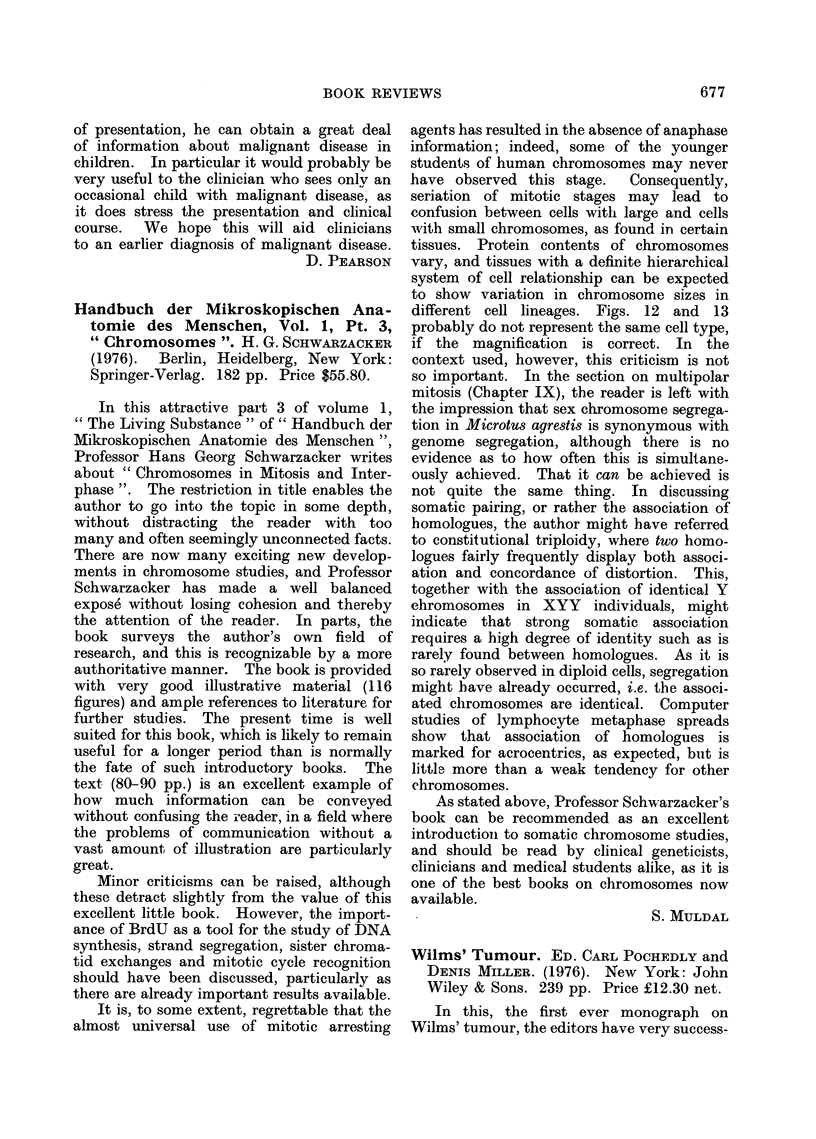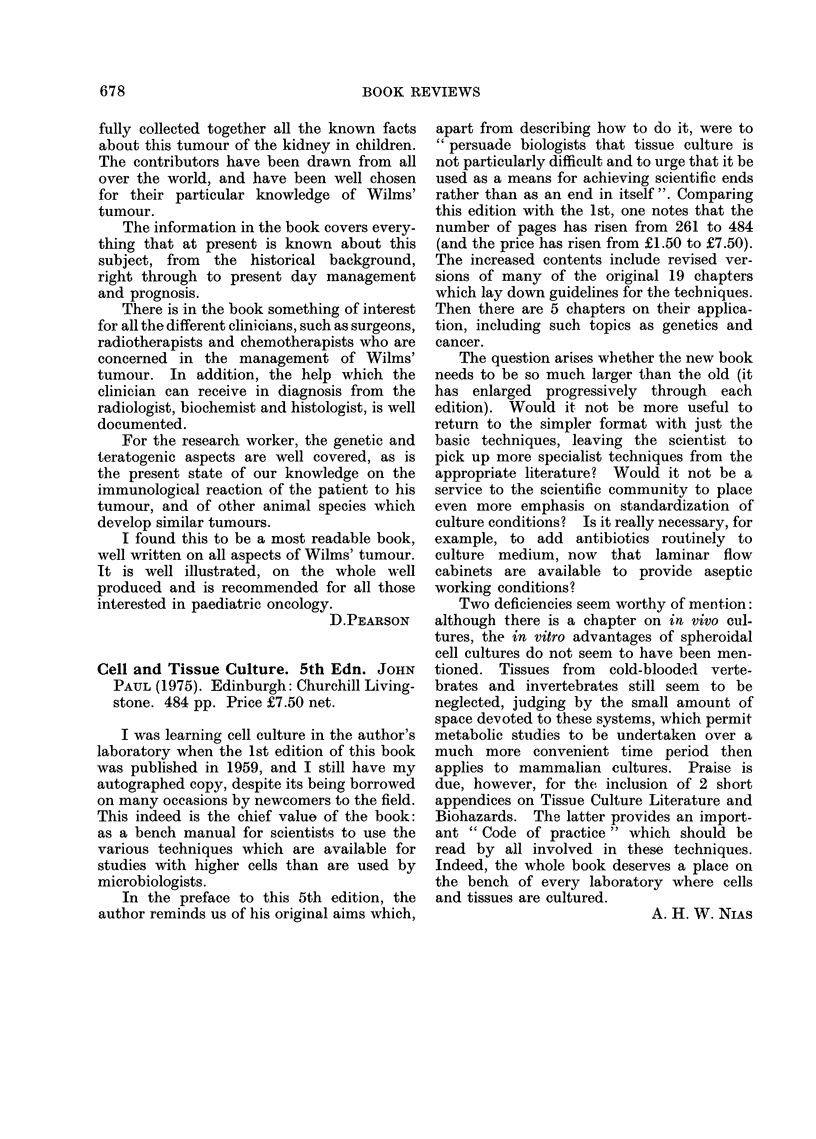# Wilms' Tumour

**Published:** 1976-12

**Authors:** D. Pearson


**Wilms' Tumour.** ED. CARL POCHEDLY and DENIS MILLER. (1976). New York: John Wiley & Sons. 239 pp. Price £12.30 net.

In this, the first ever monograph on Wilms' tumour, the editors have very successfully collected together all the known facts about this tumour of the kidney in children. The contributors have been drawn from all over the world, and have been well chosen for their particular knowledge of Wilms' tumour.

The information in the book covers everything that at present is known about this subject, from the historical background, right through to present day management and prognosis.

There is in the book something of interest for all the different clinicians, such as surgeons, radiotherapists and chemotherapists who are concerned in the management of Wilms' tumour. In addition, the help which the clinician can receive in diagnosis from the radiologist, biochemist and histologist, is well documented.

For the research worker, the genetic and teratogenic aspects are well covered, as is the present state of our knowledge on the immunological reaction of the patient to his tumour, and of other animal species which develop similar tumours.

I found this to be a most readable book, well written on all aspects of Wilms' tumour. It is well illustrated, on the whole well produced and is recommended for all those interested in paediatric oncology.